# Don’t Bathe after Meals

**Published:** 1881-07

**Authors:** 


### EXCERPTA.

Don't Bathe After Meals.—All writers on hygiene very properly discountenance bathing after meals, at least until the digestive act is fairly accomplished. The danger of disregarding this advice is illustrated by two nearly parallel cases, reported by Dr. Naegli, in the *Swiss Medical Journal:* A boy of fourteen ate a hearty meal and immediately went into the water. While swimming he suddenly gave a cry and sank. Though quickly raised to the shore the careful employment of usual resuscitative means failed. Anticipating possible obstruction the windpipe was opened and found to contain some articles of food. Before these were entirely removed the boy died. Post mortem examination revealed partial destruction of the stomach, portions of which were found in the trachea and air-tubes. The other case had a parallel history.

